# Microbiological and Chemical Assessment of Wastewater Discharged by Infiltration Trenches in Fractured and Karstified Limestone (SCA.Re.S. Project 2019–2020)

**DOI:** 10.3390/pathogens9121010

**Published:** 2020-11-30

**Authors:** Maria Teresa Montagna, Osvalda De Giglio, Carla Calia, Chrysovalentinos Pousis, Francesco Triggiano, Sapia Murgolo, Cristina De Ceglie, Francesco Bagordo, Francesca Apollonio, Giusy Diella, Marcella Narracci, Maria Immacolata Acquaviva, Giusy Bonanno Ferraro, Pamela Mancini, Carolina Veneri, Silvia Brigida, Tiziana Grassi, Antonella De Donno, Claudio Di Iaconi, Maria Clementina Caputo, Rosa Anna Cavallo, Giuseppina La Rosa, Giuseppe Mascolo

**Affiliations:** 1 Department of Biomedical Science and Human Oncology, University of Bari Aldo Moro, Piazza G. Cesare 11, 70124 Bari, Italy; mariateresa.montagna@uniba.it (M.T.M.); carla.calia@uniba.it (C.C.); vpousis@gmail.com (C.P.); francesco.triggiano@uniba.it (F.T.); francesca.apo@libero.it (F.A.); giusy.diella@uniba.it (G.D.); 2 National Research Council (CNR), Water Research Institute (IRSA), via F. De Blasio, 5, 70132 Bari, Italy; sapia.murgolo@ba.irsa.cnr.it (S.M.); cristina.deceglie@ba.irsa.cnr.it (C.D.C.); silvia.brigida@ba.irsa.cnr.it (S.B.); claudio.diiaconi@ba.irsa.cnr.it (C.D.I.); maria.caputo@ba.irsa.cnr.it (M.C.C.); giuseppe.mascolo@ba.irsa.cnr.it (G.M.); 3 Laboratory of Hygiene, Department of Biological and Environmental Sciences and Technologies, University of Salento, via Monteroni, 165, 73100 Lecce, Italy; francesco.bagordo@unisalento.it (F.B.); tiziana.grassi@unisalento.it (T.G.); antonella.dedonno@unisalento.it (A.D.D.); 4 National Research Council (CNR), Water Research Institute (IRSA), S.S. di Taranto, via Roma 3, 74123 Taranto, Italy; marcella.narracci@irsa.cnr.it (M.N.); maria.acquaviva@irsa.cnr.it (M.I.A.); rosanna.cavallo@irsa.cnr.it (R.A.C.); 5 Department of Environment and Health, Istituto Superiore di Sanità, 00161 Rome, Italy; giusy.bonannoferraro@iss.it (G.B.F.); pamela.mancini@iss.it (P.M.); carolina.veneri@guest.iss.it (C.V.); giuseppina.larosa@iss.it (G.L.R.)

**Keywords:** wastewater, groundwater, bacteria, virus, contaminants of emerging concern

## Abstract

This study investigated the environmental contamination of groundwater as a consequence of the discharge of treated wastewater into the soil. The investigation focused on a wastewater treatment plant located in an area fractured by karst in the Salento peninsula (Apulia, Italy). Water samples were collected at four sites (raw wastewater, treated wastewater, infiltration trench, and monitoring well), monthly from May to December 2019 (with the exception of August), and were tested for (1) panel of bacteria; (2) enteric viruses; and (3) chemical substances. A gradual reduction in the concentration of bacteria, viruses and contaminants of emerging concern was observed across the profile of soil fissured by karst. All monitored bacteria were absent from the monitoring well, except for *Pseudomonas aeruginosa*. Pepper mild mottle virus and adenovirus were detected at all sampling sites. Personal care products and X-ray contrast media showed the greatest decrease in concentration from infiltration trench to the monitoring well, while the highest residual concentrations in the monitoring well were found for anticonvulsants (78.5%), antimicrobials (41.3%), and antipsychotic drugs (38.6%). Our results show that parameters provided by current law may not always be sufficient to evaluate the sanitary risk relating to the discharge of treated wastewater to the soil.

## 1. Introduction

The increasing use of water resources for domestic activities, agricultural practices, and industrial processes results in the release of contaminants (e.g., bacteria, viruses, and protozoa) from wastewater treatment plants (WWTPs), with consequences for both the purification of sewage and the subsequent water reuse. The contaminants also include organic micropollutants, such as pharmaceuticals, endocrine disruptors, personal care products, pesticides, and various industrial compounds. Such contaminants are partially or completely resistant to removal in conventional WWTPs, which can lead to the formation of disinfection by-products [1]. There is much interest in the development of innovative treatments to efficiently remove a large range of organic micropollutants [2]. Such micropollutants have been detected in different aqueous compartments (e.g., WWTP effluent, surface water, and groundwater) at concentrations ranging from ng to μg per L, and their presence can cause long-term effects on both aquatic ecosystems and human health [3,4]. Some of these pollutants are classified as contaminants of emerging concern (CECs); that is, they are not currently included in any environmental regulation. The Directive 2013/39/EU has proposed a first Watch List, published in the Decision 2015/495/EU [5], that lists 17 CECs for European Union-wide monitoring. This Watch List was recently revised by the Decision (EU) 2018/840.

In many geographical areas, groundwater is the most valuable natural water resource for human needs. The natural composition of groundwater is related to the physical and chemical characteristics of the rocks that host the groundwater, the aquifer recharge because of the infiltration process through the vadose zone, and the flow of the groundwater [6]. Atmospheric pollutants, chemical products, and biological waste released directly into soil can be transported by water to reach the groundwater. Furthermore, because human settlements and industries are growing rapidly, untreated domestic sewage is sometimes drainaged into rivers, lakes, or seas, making these waters unsuitable for human use. Therefore, although one of the purposes of WWTPs is to contain the spread of pathogenic microorganisms, cases of contamination due to discharge of raw or inadequately treated wastewater are frequently reported [7,8,9,10,11,12].

In Italy, the Legislative Decree No. 152 of April 3, 2006 [13] promotes human quality of life, to be achieved through the safeguarding of environmental conditions, the sustainable use of natural resources, and the adoption of preventive measures to avoid the pollution of water bodies through inadequate water purification systems. In particular, this decree establishes the emission limits for urban and industrial wastewater discharges onto soil, with particular reference to chemical parameters. Among the microbiological parameters, only the monitoring of *Escherichia coli* (<5000 colony forming units (CFU)/100 mL) is required.

In the Apulia region in Southern Italy, the Apulian Aqueduct (AQP) [14] provides regional water supply and manages 184 WWTPs. More than half of the plants are located in the Salento area in southern Apulia and release the effluent directly onto the soil, with subsequent infiltration underground. In recent years, our group has carried out two studies on the entire region to evaluate the microbiological pollution of groundwater based on the type of aquifer [15,16]. These studies highlighted widespread fecal contamination in the Salento groundwater, which is the only water resource available to satisfy all human requirements. The use of such water resources can be severely limited, or even precluded, because of such pollution [17,18]. On the basis of these data, the Evaluation of Sanitary Risk Related to the Discharge of Wastewater to the Ground (SCA.Re.S) project was initiated by the Apulia Regional Government in 2019 to evaluate, through a multidisciplinary approach, the risk of environmental contamination deriving from the release of treated wastewater onto the soil and subsequent underground infiltration. Specifically, the SCA.Re.S project aimed: 1) to study the quality of the treated wastewater from two WWTPs that spill into a karst-fractured aquifer (WWTP-K) and a porous aquifer (WWTP-P), and 2) to verify if the different soils can influence the quality of the water downstream of the WWTP that reaches the aquifers.

In this paper, we report the results of the study relating to the WWTP located in the karst-fractured area.

## 2. Results and Discussion

### 2.1. Bacterial Detection

Table 1 shows the results from the microbiological analysis of the treated wastewater, infiltration trench, and monitoring well, expressed as average concentrations. *Salmonella* spp. were not present. The results were positive for *E.coli*, *Enterococcus* spp., and *C. perfringens* in both the treated wastewater and the infiltration trench, with the mean loads always below the limit allowed by Lgs.D. 152/06. The mean values of the *E.coli*, *Enterococcus* spp., and *C. perfringens* concentrations were greater in the infiltration trench, compared with the treated wastewater, but were < 1 CFU/100 mL in monitoring well. In contrast, *P. aeruginosa* was observed to have an increased load in the water samples from the monitoring well, compared with the other two sampling sites.

Wastewater can carry many opportunistic pathogens (e.g., *Enterobacter cloacae*, *Enterococcus faecalis, Escherichia coli, Klebsiella pneumoniae,* or *Pseudomonas aeruginosa*), which can cause different systemic infections, especially among people with a weakened immune system [19]. While the levels of *E. coli* are an essential parameter for assessing the quality of treated wastewater, *Enterococcus* spp. and *C. perfringens* can survive more easily in the environment, including in water and soil, so their presence can indicate inadequate treatment. In particular, the presence of *C. perfringens*, because of its considerable ability to survive in the form of spores, represents a particularly valid parameter for determining the quality of environmental matrices in which other fecal contamination indicators are more easily eliminated. This bacterium is the main etiological agent of myonecreosis of connective tissues [20] and may be also responsible for food poisoning and diarrhea [21]. *P. aeruginosa* is a free-living bacterium found primarily in soil, seawater, and natural waters (lakes and rivers) [22]. During periods of heavy rain, *P. aeruginosa* can infiltrate into groundwater and contaminate aquifers. The ability of *P. aeruginosa* to adapt to a wide range of habitats accounts for its ubiquitous presence in the environment and important impact on ecology and agriculture. This microorganism ranks among the most important human opportunistic pathogens, being responsible for eye and skin diseases, pneumonia, and sepsis; infections are often severe and associated with high mortality, especially if the bacterial strains are multi (drug)-resistant [23]. Therefore, the presence of *P. aeruginosa* in water used for irrigation can be a public health problem.

Only two samples from the infiltration trench gave positive results for *Vibrio* spp., showing the presence of *V. litoralis* (28.7%), *V. salmonicida* (23.9%), and *V. furnisii* (47.4%) in the first sample and *V. furnisii* (67%) and *V. salmonicida* (33%) in the second sample. A feature of the karst aquifer studied is that seawater encroaches into the fresh water, creating an underground connection between the waters of the Ionian and Adriatic seas and making groundwater along the coast with higher salinity. Vibrio species are largely halophilic, but depending on their sodium chloride requirements, a few species are non-halophilic also. These bacteria are common inhabitants of coastal marine ecosystems [24], but also have a well-documented adaptability to adverse conditions that allows their presence in other environments, such as rivers, fresh water, pond water, tap water, and groundwater [25]. 

Among the *Vibrio* spp. detected in this study, only *V. furnissii* is considered pathogenic in humans, and can give rise to human gastroenteritis and extra-intestinal manifestations [26].

Carbonate rocks, in which the Apulia territory is rich, being more soluble than other rocks are involved in the processes of alteration, erosion, and dissolution that can create spectacular morphologies such as sinkholes, sinking streams, closed depressions, underground infiltrations, and caves. Thus, a strong interaction between the surface water and groundwater characterizes the karst landscape. Because of the very high infiltration rates in karst areas, surface flow is rare, compared with non-karst areas, and groundwater is the main source of water for human requirements, including irrigation and industry. For this reason, it is very important to preserve the quality of groundwater, which is sometimes of poor hygienic quality, even though self-purification via filtration can occur during the infiltration process. Generally, in karst areas or in fractured aquifers, a series of unfavorable characteristics, such as rapid flow rates and lack of filtration and self-purification can reduce the effect of the dilution or dispersion of pollutants.

Overall in the present study, we observed an increased concentration of bacteria in the water samples of infiltration trench and a reduction across the karst-fissured soil profile until below limit detection (<1 CFU/100mL) in the monitoring well. Regarding the first result, it was probably due to the environmental conditions of treated wastewater infiltration trench (temperature, humidity, nutritional factors, stagnation, etc.) favorable to the proliferation of bacteria [27]. Conversely, different soil properties could be probably involved in the reduction of the presence of bacteria in the monitoring well, such as soil pores, and bacterial adsorption onto soil particles [28,29], although it is known that sandy or gravel aquifers hinder the spread of microorganisms, while karst-fissured aquifers are more vulnerable because flow can be relatively rapid in these aquifers [30,31]. Furthermore, the presence of channels originating from plant root systems and the burrows of some organisms, such as earthworms, can strongly influence the vertical migration of pathogens through the soil profile [32]. Additionally, we hypothesized that reductions in microbial and chemical pollution could also be caused by natural degradation over time, photodegradation, and ingestion by multicellular or unicellular organisms in the soil or monitoring well, or both [33,34,35,36].

### 2.2. Enteric Virus Detection

The results obtained by nested reverse transcription (RT)-polymerase chain reaction (PCR) are summarized in Table 2. All the samples tested positive for at least one viral pathogen. The enteric viruses, NoV, RoV, EV, AdV, and HAV and HEV, were selected for this study because they are the most common viral agents detected in water environments worldwide, causing gastroenteritis through ingestion of contaminated water or fruit and vegetables [10,16,37].

Overall, the occurrence of the enteric viruses decreased along the pathway of the environmental purification and disposal of wastewater. NoV was present in all samples from raw sewage (two samples contained NoV belonging to GI genogroup and five samples contained NoV of GII genogroup).

EVs were present only in the raw sewage samples. HEV was only detected in one infiltration trench sample.

HAV and RoV were not detected in any of the samples analyzed, in accord with previous studies in the same area [16,18]. In the Apulia region, following a large epidemic in 1998, a vaccination program for hepatitis A was introduced for toddlers and preadolescents. Consequently, the incidence of this disease dramatically decreased between 2005 and 2014, which could explain our study results [16,38]. Rotaviruses are a major cause of hospitalization in children in Apulia, especially in the Salento area; however, while these viruses have been detected in raw and treated sewage and surface water [39,40], there is little evidence of groundwater contamination [18].

PMMoV, a plant virus infecting peppers, is excreted in the human feces of healthy populations at high concentrations, and its presence in water environments is associated with dietary intake (consumption of peppers and processed products, such as hot sauce and curry). PMMoV does not replicate in human intestines so does not cause human infections [41]. The PMMoV has been proposed as an indicator for water pollution and to verify the efficiency of water treatment processes [42,43,44,45].

In the present study, PMMoV was detected at all the sampling sites and in all samples from the infiltration trench. Our results highlighted that PMMoV appears to be more persistent in water environments than other viruses [46]. Moreover, in agreement with other studies [47], our results showed that wastewater treatment does not reduce the incidence of PMMoV, which appeared to follow a different trend than the other viruses, probably because of its natural predominance in environmental habitats.

### 2.3. Detection of Chemicals

#### 2.3.1. Chemical-Physical Parameters

Table 3 shows the mean values of the chemical-physical parameters recorded at the different sampling sites. All the values of the parameters for the treated wastewater were below the limit allowed by L.Dgs 152/06. Except for the total nitrogen (TN), there was a reduction in the value of each parameter in the monitoring well, compared with that in the raw sewage.

#### 2.3.2. Contaminants of Emerging Concern

A total of 37 target emerging contaminants and their degradation products were detected. Table 4 shows the mean concentration and the standard deviation of each emerging contaminant and the sum (in bold) of the substances grouped by pharmacological category in the treated wastewater, infiltration trench, and monitoring well.

In the monitoring well, the highest concentrations were observed for olmesartan (1.36 μg/L) and irbesartan 446 (0.24 μg/L) in the beta-adrenoceptor blocking agents; carbamazepine (0.49 μg/L) and lamotrigine (0.33 μg/L) in the anticonvulsants; 2-ethyl-1.5-dimethyl-3.3-diphenylpyrrolidine (EDDP) (0.25 μg/L) and sulpiride (0.14 μg/L) in the antipsychotic drugs, and fluconazole (0.49 μg/L) in the antimicrobials.

Antipsychotic drugs and their metabolites (in particular, EDDP, a metabolite of methadone) were found at higher concentrations in the present study than in other studies that were performed in deep water [48].

**Figure 1:**
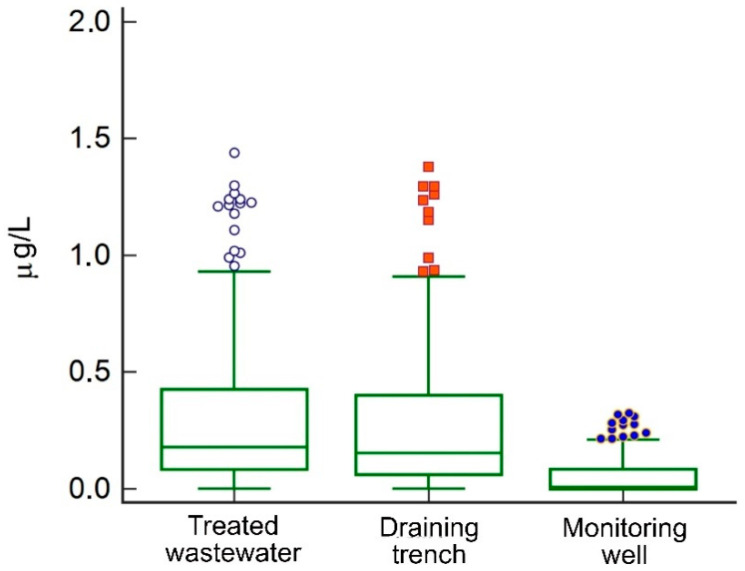
Distribution of the concentration values of the substances in the treated wastewater, infiltration trench, and monitoring well. Far out values, identified by the Tukey test, were eliminated.

A total of 750 determinations, 250 at each investigated site, were carried out during the monitoring period. In Figure 1, a box and whisker plot shows the distribution of the concentration values of the substances in the treated wastewater (highest value 25.4 μg/L, arithmetic mean 0.74 μg/L, median 0.17 μg/L, SD 2.29 μg/L), infiltration trench (highest value 28.8 μg/L, arithmetic mean 0.78 μg/L, median 0.20 μg/L, SD 2.15 μg/L), and monitoring well (highest value 2.04 μg/L, arithmetic mean 0.12 μg/L, median 0.02 μg/L, SD 0.26 μg/L), after the elimination of the far out values identified by the Tukey test. The data did not follow a normal distribution in any of the groups.

**Figure 2:**
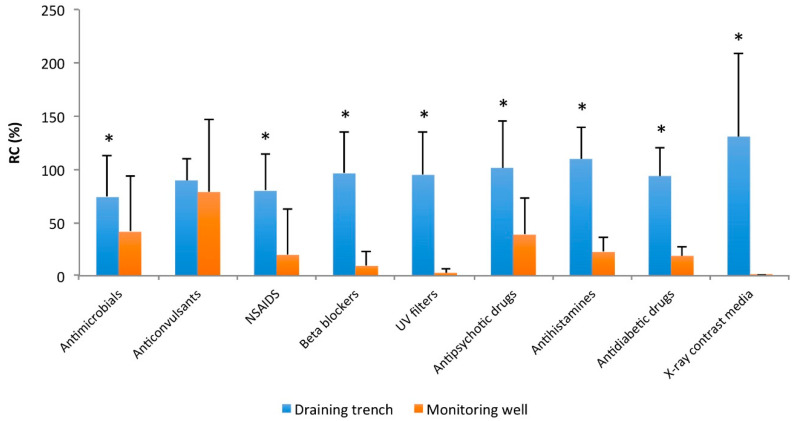
Average residual concentration (RC) and standard deviation of substances in the infiltration trench and monitoring well grouped by pharmacological category (* *p* < 0.05 calculated by Student’s *t*-test; ^nonsteroidal anti-inflammatory drugs).

On average, the highest residual concentration (RC) in the infiltration trench was detected for X-ray contrast media (130.6%) and antihistaminic drugs (109.6%), whereas the lowest RC was found for antimicrobials (73.8%) and anti-inflammatory substances (79.7%). In the monitoring well, the highest RC was detected for anticonvulsants (78.5%), followed by antimicrobials (41.3%) and antipsychotic drugs (38.6%), whereas the lowest RC values were detected for UV filters (2.74%) and X-ray contrast media (0.05%) (Figure 2).

The concentrations of all the identified compounds underwent a significant decrease (*p* < 0.05) during the filtration through the soil layers after the dispersion of the treated wastewater in the infiltration trenches, except for that of the anticonvulsant drugs. In particular, personal care products, UV filters, and X-ray contrast media were the contaminants that showed the greatest decrease in concentration from the infiltration trench to the monitoring well.

**Figure 3:**
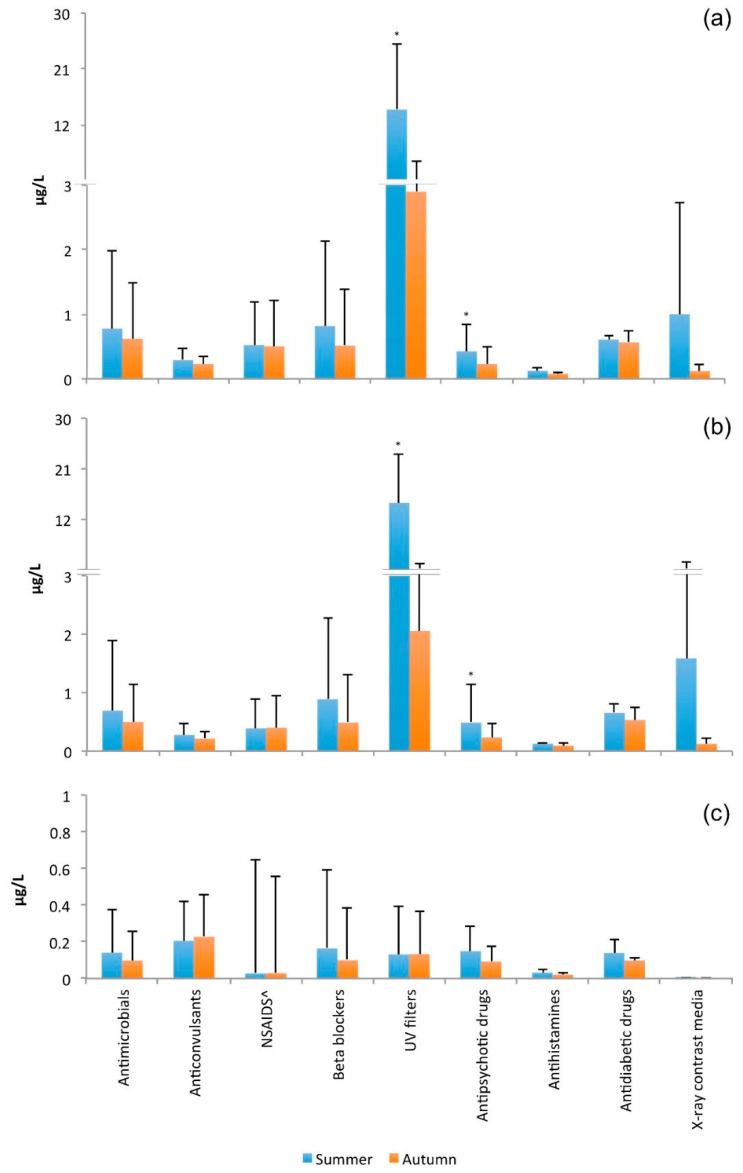
Average seasonal concentrations and standard deviation of emerging pollutants, grouped by pharmacological category, in the treated sewage (**a**), draining trench (**b**), and monitoring well (**c**). * *p* < 0.05 calculated by the Kruskal–Wallis test.

Figure 3 shows the average seasonal concentrations of emerging pollutants, grouped by pharmacological category, in the treated wastewater (a), draining trench (b), and monitoring well (c).

The statistical analysis revealed that the concentration of some substances (in particular, UV filters and antipsychotic drugs) in the treated wastewater and in the draining trench was significantly decreased (*p* < 0.05) from summer to autumn. This result was probably because there is generally less use of these substances in autumn (e.g., UV filters) or because of meteorological factors, such as evaporation caused by higher temperatures in summer and dilution caused by more abundant rainfall during autumn, as previously described [49,50]. The meteorological data collected by the Regional Agency for Environmental Protection (ARPA Puglia) [51] showed an average temperature in the study area of 26.1°C in summer and 17.7 °C in autumn and the total rainfall was 99.8 mm in summer and 286.6 mm in autumn. This seasonal effect was not observed in the groundwater, where the concentrations of contaminants were not significantly different between the two seasons.

## 3. Materials and Methods 

### 3.1. Study Area

The Apulia region covers approximately 20,000 km^2^ and has four million inhabitants. The region spans approximately 350 km between the Adriatic and Ionian Seas, and has extensive coastal development along its 800 km of coastline. The area has a typical Mediterranean climate, with irregular annual rainfall and is characterized by few surface water bodies because of its geological characteristics, which predominantly feature fractured carbonate rocks locally subjected to a continuous karstification process.

**Figure 4:**
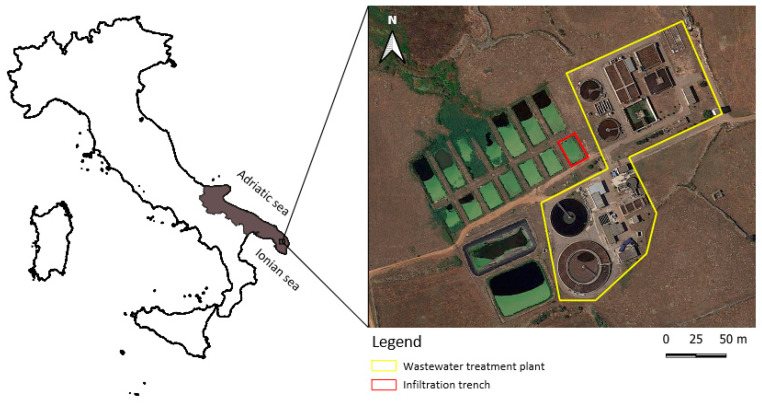
WWTP-K in Salento peninsula, Apulia, Italy.

The study area is located in the inland region of the Salento peninsula. This region is a flat area, 200 m above sea level, extending over 150 km between the Ionian and Adriatic seas (Figure 4).

The Salento peninsula is a Mesozoic carbonate succession of carbonate and clayey-sandy deposits. The carbonate basement consists of limestone, dolomitic limestone, and dolostone with sub-horizontal layers ascribable to the Calcari di Altamura Formation (Upper Cretaceous). The main groundwater reservoir is present in this formation, whereas tertiary and quaternary deposits have locally hosted, shallow, and limited aquifers. The Cretaceous carbonate rocks are characterized by a high permeability, which is the result of tectonics that influenced both the type and degree of fracturing of the rock and the evolution of the karst processes. This tectonic influence explains the coexistence of rocky horizons with different types and degrees of permeability, and with discontinuous and variable thicknesses, which considerably affects the permeability along the horizontal and vertical directions [52], and therefore the aquifer vulnerability.

Groundwater pollution caused by human activities, such as wastewater disposal, may easily compromise this valuable natural water resource because of the high vulnerability of the karst aquifers [53,54].

### 3.2. Sample Collection

The studied WWTP-K (Figure 4) is a municipal depuration plant that performs treatment processes, including grilling and sand separation, denitrification, biological nitrification, and secondary sedimentation. Successively, wastewater is subjected to chiariflocculation and disinfection with chlorine and UV rays. This depuration plant is equipped with an infiltration-trench system used for disposing treated wastewater to the soil, with a monitoring well located inside the WWTP-K area (well depth = 90 m, distance from the effluent duct of the plant = 63 m) used routinely by the AQP to monitor the plant activity.

From May to December 2019, with the exception of August, 28 water samples from raw sewage (n = 7), treated wastewater (n = 7), the infiltration trench (n = 7), and the monitoring well (n = 7) were collected monthly between 8:00 am and 11:00 am. Sterile containers were used for the sampling, which was performed under calm atmospheric conditions, with no rain. Microbiological, chemical-physical parameters and contaminants of emerging concern beyond those required by Italian law [13] were determined to evaluate the quality of the water downstream of the WWTP that reaches the aquifers.

### 3.3. Detection of Bacteria 

Two liters of treated wastewater, infiltration trench water, and monitoring well water were collected during each sampling for the detection of bacteria. Samples were transported in a refrigerator (+4 °C) and processed within 5 h. Water samples were tested for indicators of faecal contamination (*E.coli*, *Salmonella* spp., *Enterococcus* spp, *P. aeruginosa, C. perfringens*, and *Vibrio* spp.).

#### 3.3.1. *Escherichia coli*

Each sample (100 mL) was filtered through a cellulose ester membrane filter (47 mm Ø and 0.45 µm-pore size; Millipore Corporation, Bedford, MA, USA) placed on plates containing Chromogenic Coliform Agar (Biolife Italiana Srl, Milan, Italy). After incubation at 36 ± 2 °C for 24 ± 2 h, the blue-violet colonies were identified as *E. coli* [55]. The results were reported as CFU/100 mL.

#### 3.3.2. *Salmonella* spp.

Each sample (1000 mL) was filtered through cellulose nitrate membrane filters (47 mm Ø and 0.45-mm pore size; Millipore, Milan, Italy). The membrane was then immersed in Buffered Peptone Water (BPW) (Biolife Italiana srl, Milan, Italy) and incubated for 18–24 h at 36 ± 1 °C. Subsequently, a 0.1-mL aliquot of culture was transferred to 10 mL of Rappaport Vassiliadis broth (Microbiol & C. s.n.c, Uta, Italy) and incubated for 24 + 24 h at 41.5 °C. The broth was then streaked after 24 and 48 h on xylose lysine deoxycholate agar plates (Biolife Italiana srl) and Hektoen Enteric Agar (Merck, Darmstadt, Germany), respectively. After 24 h at 36 ± 1 °C, colonies with typical morphology were streaked on Tryptic Soy Agar plates (Biolife Italiana srl), incubated at 36 ± 1 °C for 24 h and subjected to biochemical confirmation tests (API 20E, Biomèrieux, Marcy l’Etoile, France). Finally, typing through specific serological tests [56] was performed.

#### 3.3.3. *Enterococcus* spp.

A 100-mL aliquot of each sample was filtered through a cellulose ester membrane (47 mm Ø and 0.45-µm pore size; Millipore, Milan, Italy). The membrane was placed over a Slanetz and Bartley agar medium (Biolife Italiana srl, Milan, Italy) and incubated at 36 ± 1 °C for 48 h. The colonies ranged in color from pink to dark red and brown, but only catalase- and esculin hydrolysis-positive colonies were considered to be enterococci [57]. The results were reported as CFU/100 mL.

#### 3.3.4. *Pseudomonas aeruginosa*

Each sample (250 mL) was filtered through a cellulose ester membrane (47 mm Ø and 0.45-µm pore size; Millipore, Milan, Italy). The membrane was placed on a plate containing Pseudomonas selective agar supplemented with cetrimide (0.20 g) and nalidixic acid (15 mg) (Microbiol, Cagliari, Italy) and incubated at 36 ± 2 °C for 44 ± 4 h. Blue-green pyocyanin producing colonies were directly confirmed to be *P. aeruginosa* [58]. The results were reported as CFU/250 mL.

#### 3.3.5. *Clostridium perfringens*

Each sample (100 mL) was pretreated at 75 ± 5 °C for 15 ± 1 min in a water bath. After filtration, the membrane filter (47 mm Ø and 0.45-µm pore size; Millipore, Milan, Italy) was placed on Triptosio Solfito Cicloserina Agar (Biolife Italiana srl, Milan, Italy) plates, and incubated at 44 °C for 24 h in an anaerobic atmosphere generated by GasPak EZ Gas Generating Pouch Systems (BD Diagnostics, UK). Black colonies were considered to be spores of sulfite-reducing clostridia. The results were reported as CFU/100 mL [59].

#### 3.3.6. *Vibrio* spp.

Qualitative *Vibrio* spp. Detection was carried out by filtering 100-mL water samples through 0.45-µm pore size filters (Millipore). The membranes were incubated on thiosulfate-citrate-bile-salt (TCBS, Thermo Scientific™ Oxoid™) agar plus 2% NaCl and incubated at 37 °C for 24–48 h. The suspected colonies (yellow and green) were picked out, streaked onto nutrient agar plus 2% NaCl to obtain pure cultures, and screened using morphological, biochemical, and culture tests such as lysine and ornithine decarboxylase, growth at different NaCl concentrations and temperatures, citrate, gelatinase, glucose gas, indole, luminescence, NO_2_, ONPG, oxidase, urease, Voges Proskauer, d-glucose growth, L-arabinose growth, acid from arabinose, mannitol, D-mannose, rhamnose, salicin, sucrose, O/129 10 µg and 150 µg [60].

To enumerate the Vibrio spp., each sample was filtered on 0.45-µm pore size filters and the filter disks were aseptically placed onto TCBS agar. The mean values for three replicate samples were determined for both qualitative and quantitative determination. After incubation for 48 h, the results were reported as CFU/100 mL.

### 3.4. Enteric Virus Detection 

All the water samples were tested for a panel of enteric viruses: enterovirus (EV), adenovirus (AdV), norovirus (NoV), hepatitis A and E (HAV and HEV), rotavirus (RoV), and pepper mild mottle virus (PMMoV).

One protocol was used to concentrate raw sewage [61] and a second protocol was used for all other water samples (treated wastewater, infiltration trench, and monitoring well water) [62]. Before concentration, a murine norovirus (MNV-1) was added to all samples as a sample process control (1 mL, 8.81 × 10^4^ genome copies).

#### 3.4.1. Raw Sewage Samples

Raw sewage samples (250 mL) were concentrated using the two-phase (polyethylene glycol (PEG)-dextran) separation method recommended by the WHO guidelines for environmental surveillance of poliovirus circulation [54]. Viral nucleic acid extraction was carried out on chloroform-treated samples (5 mL) using the NucliSENS MiniMag (bioMerieux, Marcy l’Etoile, France) semi-automated extraction system, in accordance with the manufacturer’s instructions. Eluted RNA (100 µL) was stored in aliquots at −80 °C until molecular analysis.

#### 3.4.2. Treated Wastewater, Infiltration Trench, and Monitoring Well Samples

Samples of treated wastewater (40 L), infiltration trench water (60 L), and monitoring well water (1000 L) were filtrated by Nanoceram electropositive cartridges (Argonide Corporation, Sanford, FL, USA) following the virus adsorption–elution (VIRADEL) technique [62] After filtration, each cartridge was eluted with 400 mL of 3% beef extract (pH 9.5) by direct contact in the housing, which was agitated on an orbital shaker for 20 min. The cartridge was then removed and the eluate was neutralized with 1.2 M NaOH. The secondary concentration step was performed by PEG precipitation. PEG 6000 and NaCl were added to reach final concentrations of 10% and 1.6% *w*/*v*, respectively. The suspension was incubated at 4 °C overnight and then centrifuged (8650× *g*) at the same temperature. The final pellet was dissolved in 10 mL of phosphate-buffered saline (PBS) pH 7.4 and stored at −20 °C for nucleic acid extraction. Nucleic acid extraction was performed as for raw sewage.

#### 3.4.3. Nested PCR Assays and Sequencing

A nested reverse transcription (RT)-polymerase chain reaction (PCR) assay was used for the detection of enteric viruses. The primers and conditions used in this study are listed in Table 5. For RNA viruses, the cDNA synthesis was performed with the SuperScript^®^ IV First-Strand Synthesis System (Invitrogen). PCR reactions for EV, AdV, HAV, HEV, RoV, and PMMoV were performed using the MyTaqTM red mix kit (Bioline) in a 25-μL mixture containing 12.5 μL of mix, 1 μL (10 pmol) of each primer, and 2 μL of the extracted genome. In the second amplification cycle, 1 μL of PCR product underwent the nested amplification. Detection of norovirus genogroups I and II (NoV GI and GII) was performed using the PlatinumTM Green Hot Start PCR Master Mix kit (Invitrogen), using the amplification conditions according to the manufacturer’s instructions except for the annealing temperatures, which are reported in Table 5. PCR was performed in a T100 thermal cycler (BioRad, Hercules, CA, USA). Amplicons of the nested PCR were visualized by gel electrophoresis on 2% agarose gels containing GelRed staining (Biotium, CA, USA), then purified using a Montage PCRm96 Microwell Filter Plate (Millipore, MA, USA) and sequenced on both strands by Bio-Fab Research (Rome, Italy). Sequences were compared with available sequences in the GenBank database using BLAST [63].

### 3.5. Chemical-Physical Parameters

Each sample was analyzed for the physico-chemical parameters provided by Lgs D. 152/06 [13]. The temperature, pH and conductivity were measured in situ with a multiparametric probe (WTW MultiLine P4) according to potentiometric [74] and conductivity [75] methods. Total suspended solids (TSS) were determined by filtration method through glass fiber filters [76]; biochemical oxygen demand (BOD5) by respirometric method [77] and chemical oxygen demand (COD) was detected using the sealed tube method according to International Standard Organization [78]. Total nitrogen (TN) was measured by UV spectrometry after oxidative digestion with sodium persulfate using an actuator that operates in a coordinated analytical sequence [79]. The measure of nitrates (NO3) was performed through the determination of dissolved anions by liquid phase ion chromatography [80], and total phosphorus (TP) by colorimetric method of ammonium phosphoantimonylmolybdate after coordinated analytical sequence [81].

### 3.6. Detection of Contaminants of Emerging Concern

The analytical standards used to prepare the stock solutions required for qualitative and quantitative investigation (purity range of 96.0–99.0%) were purchased from LabService Analytica (Anzola Emilia, Bologna, Italy). A stock solution of an internal standard, carbamazepine D10 (10 mg/L in methanol) was used to spike all samples prior to the analytical determination, at a final concentration of 10 μg/L. All the solvents used for chromatographic analyses for preparing standard solutions—acetonitrile, methanol, and formic acid—were of ultra-performance liquid chromatography grade, and ultrapure water was obtained by a Milli-Q Gradient A-10 system (Millipore, Burlington, VT, USA).

The occurrence of contaminants of emerging concern in the treated wastewater, infiltration trench and monitoring well samples was investigated using an analytical detection method that was developed to have increased specificity and sensitivity for the analysis of organic compounds, which have different chemical properties and are present in trace quantities in the investigated matrices. An ultra-high pressure liquid chromatograph (Ultimate 3000 System, Thermo Fisher Scientific) interfaced with a high-resolution tandem mass spectrometer (TripleTOF^®^ 5600+ System, AB Sciex), with a duo-spray ion source operated in electrospray ionization (ESI) positive mode, was employed. All mass spectrometry (MS) analyses were performed using an acquisition method based on double experiments, time of flight (TOF)-MS/information dependent acquisition (IDA) in the mass scan range 50-1000 Da. An accumulation time of 0.200 s was applied for full-scan survey TOF-MS and 0.07 s for IDA scans. The MS interface operating parameters (arbitrary units) were as follows: nebulizer gas 35, turbo gas 45, curtain gas 20, ion spray voltage 5500 V, declustering potential 80 V, temperature 500 °C. The collision energy voltage was set at 35 with a collision energy spread of 15.

To extend the range of compounds identified at very low detection limits, 2000 μL of each analyzed sample, previously filtered with a 0.20-μm nylon filter, was injected using an online solid phase extraction (SPE) method by a 10-port, 2 position (1-2, 1-10) method.

For analyte enrichment a Hypersil GOLDaQ column (20 × 2.1 mm, 5 µm) was used, operating at a flow rate of 0.250 mL/min using 100% water. The analytes retained on the SPE column were then separated, using an analytical column (Waters BEH C18 column (150 × 2.1 mm, 1.7 µm)) operating at a flow rate of 0.200 mL/min at a temperature of 40 °C and eluted with a binary gradient consisting of water (A) and acetonitrile (B) both with 0.1% formic acid as follows: 9 min 2% B, 10 min 20% B, 24.5 min 100% B, 33.5 min 100% B, 33.7 min 2% and equilibration for 10 min before the next run. To make the analytical method more robust, the internal standard carbamazepine D10 was added to each sample prior to the analytical determination. In this way, it was possible to take into consideration the relative reduction in the signal intensity because of the matrix effect of each sample.

All the raw data obtained by the high-resolution MS analysis were processed using the AB Sciex software, SciexOS 1.2, PeakView 2.2, MasterView 1.1, and LibraryView 1.1.0. The identified compounds were regularly monitored in all the samples collected from the WWTP-K to detect any significant trends.

The analytical protocol employed for the screening of organic contaminants allowed the identification of compounds present in the investigated samples. Briefly, the AB Sciex software screened for the presence of target compounds by comparing the retention time and MS/MS fragmentation pattern of the detected compounds with those of standard compounds. A mass accuracy error (<5 ppm), an isotopic pattern fit (>90%), and the presence of matching fragments (at least three with a mass error <5 ppm), provided supporting evidence for the identification of compounds. A reference calibration curve was injected in the range 0.1–1 μg/L (0.1, 0.25, 0.50, 0.75, and 1 μg/L) to quantify all the identified compounds in the samples.

### 3.7. Statistical Analysis

The results from the analytical investigation were entered into a database and statistically processed using MedCalc Software version 12.3 (MedCalc Software bvba, Ostend, Belgium).

For each group of data, the arithmetic mean, the standard deviation, the maximum value, and the median were calculated. In particular, the average concentration of microbiological parameters was calculated by setting for negative samples a value equal to half (0.5) of the detection limit (<1 CFU/100 mL) as described in Lorimer and Kiermer, 2007 [82]. The D’agostino–Pearson normality test was used to determine if data sets were well modeled by a normal distribution, and the Tukey test was used to detect outliers. The RC of each substance in the infiltration trench and the monitoring well was calculated as the percentage of the concentration at the sampling point divided by the concentration in treated wastewater. Any significant differences between the RCs of the substances grouped by pharmacological category in the different monitoring points were determined by the Student’s *t*-test.

To determine any seasonal differences, the concentrations of the substances in each pharmacological category were grouped according to the sampling month into summer samples (samples collected in June, July, and September) and autumn samples (samples collected in October, November, and December). Group comparison was performed using the Kruskal–Wallis nonparametric test because the data did not follow a normal distribution.

## 4. Conclusions

According to previous studies [83,84], our results show that the monitoring for *E. coli* only may not always be sufficient to evaluate the sanitary risk relating to the discharge of treated wastewater to the soil. Therefore, governmental organizations should consider incorporating permissible levels for other microbiological parameters in their regulations.

Overall, the monitoring of a wide range of bacteria and viruses at sampling sites (treated sewage, infiltration trench, and monitoring well) revealed a gradual reduction in the concentration of these contaminants along the karst-fissured soil profile, until complete removal of most of the contaminants was observed in the aquifer of the monitoring well. However, *P. aeruginosa*, PMMoV, and AdV were detected in the monitoring well, demonstrating that several factors can influence the transport of microbiological contaminants through the soil, such as the type and size of the microorganism, the adsorption capacity on soil, soil pores, and soil particles, and the usual environmental habitat of the microorganism.

We hypothesized that the reduction of pollutants in the water as it moves from the wastewater treatment plant to the monitoring well could also be attributable to various other phenomena, such as natural degradation over time, photodegradation, and ingestion by unicellular or multicellular organisms in the soil or monitoring well, or both.

These findings are also supported by the concentration trends for the different CECs identified and monitored in the samples from the treated wastewater, infiltration trench, and monitoring well. The concentration of most of the compounds grouped by pharmacological category decreased during filtration through the soil layers, after the dispersion of the treated wastewater in the infiltration trenches. In particular, personal care products and X-ray contrast media were the contaminants that showed the greatest decrease in concentration from the infiltration trench to the monitoring well, whereas the highest RC was detected for anticonvulsants, followed by antimicrobials and antipsychotic drugs.

Finally, a similar study will be implemented in a wastewater treatment plant located in an area of Salento with porous geology to investigate the influence of the soil on the pollution process.

## Figures and Tables

**Table 1:** Average concentration (± standard deviation) and number of positive samples (No.) out of total samples (N.) of the microbiological parameters in the treated wastewater, infiltration trench, and monitoring well.

Water Samples	*Escherichia coli*		*Enterococcus* spp.		*Pseudomonas aeruginosa*		*Clostridium* *perfringens*		*Vibrio* spp.	
	CFU/100 mL	Positive No./N.	CFU/100 mL	Positive No./N.	CFU/250 mL	Positive No./N.	CFU/100 mL	Positive No./N.	CFU/100 mL	Positive No./N.
Treated wastewater	5.9 ± 4.6	7/7	4.9 ± 11.1	3/7	34.0 ± 88.6	1/7	5.2 ± 2.28	7/7	-	0/7
Infiltration trench	283.3 ± 512.4	7/7	118.5 ± 169.1	6/7	143.2 ± 200.7	6/7	16.2 ± 19.9	7/7	193.6 ± 469.5	2/7
Monitoring well	-	0/7	-	0/7	7990.3 ± 10,316.2	7/7	-	0/7	-	0/7

**Table 2:** PCR results for virus detection in the raw sewage, treated wastewater, infiltration trench, and monitoring well.

Water Samples	No.	Number of PCR-Positive Samples							
		PMMoV	AdV	NoV-GI	NoV-GII	EV	HEV	HAV	RoV
Raw sewage	N = b7	5	5	2	5	4	0	0	0
Treated wastewater	N = b7	6	3	0	3	0	0	0	0
Infiltration trench	N = b7	7	1	0	0	0	1	0	0
Monitoring well	N = b7	3	1	0	0	0	0	0	0

Legend: enterovirus (EV), adenovirus (AdV), norovirus (NoV), hepatitis A and E (HAV and HEV), rotavirus (RoV), and pepper mild mottle virus (PMMoV).

**Table 3:** Mean value (range) of chemical-physical parameters in the water samples from the WWTP-K.

Source	T (°C)	pH	Conductivity µS/cm	TSS * mg/L	BOD_5_ * mg/L	COD * mg/L	TN * mg/L	NO_3_ * mg/L	TP * mg/L
Raw sewage	-	7.7 (7.5–7.8)	1698.7 (1658–1860)	353 (180–500)	580 (300–620)	859 (436–900)	74.1 (47.6–79.4)	4.9 (4.5–5.1)	10.7 (7–11)
Treated wastewater	19.7 (16.1–22.3)	7.4 (7.4-7.5)	1079.5 (892–1267)	5.25 (4–7)	10.25 (7–11)	27.5 (22–36)	14.3 (7.8–27.9)	2 (2.6–3.8)	2.0 (1.4–3.0)
Infiltration trench	25.4 (22.8–30.0)	7.6 (7.4–7.9)	1294.1 (1193–1348)	0	15 (12–16)	24 (21.3–26.5)	17.56 (12.5–19.7)	2.5 (2.2–2.9)	2.5 (2.4–2.6)
Monitoring well	21.1 (20.1–22.7)	7.3 (7.1–7.8)	1162.5 (1160–1165)	0	0 (0–1)	2.5 (0–7)	6.9 (5.1–8.0)	26.4 (24–30)	0.71 (0.70–0.72)

* Total suspended solids (TSS), biochemical oxygen demand (BOD_5_), chemical oxygen demand (COD), total nitrogen (TN), nitrates (NO_3_), and total phosphorus (TP).

**Table 4:** Mean concentrations (M) (μg/L) (± standard deviation, SD) and number of positive (n.) out of total samples (N.) of each target contaminants of emerging concern (CEC) and the sum of the concentrations grouped by pharmacological category (in bold) in the treated wastewater, infiltration trench, and monitoring well.

	Treated Wastewater		Infiltration Trench		Monitoring Well	
	n./N.	M ± SD	n./N.	M ± SD	n./N.	M ± SD
**Antimicrobials**	**7/7**	**2.46 ± 1.27**	**7/7**	**1.98 ± 1.46**	**7/7**	**0.49 ± 0.11**
*Levofloxacin*	7/7	1.89 ± 1.19	7/7	1.51 ± 1.34	2/7	0.04 ± 0.01
*Azithromycin dihydrate*	7/7	1.18 ± 1.47	7/7	0.95 ± 1.05	0/7	-
*Fluconazole*	7/7	0.38 ± 0.14	7/7	0.37 ± 0.16	7/7	0.43 ± 0.11
*Clarithromycin*	7/7	0.10 ± 0.05	7/7	0.04 ± 0.02	1/7	0.02 ± 0.01
*Climbazole*	7/7	0.08 ± 0.03	7/7	0.06 ± 0.03	7/7	0.04 ± 0.05
**Anticonvulsants**	**7/7**	**1.02 ± 0.25**	**7/7**	**0.96 ± 0.29**	**7/7**	**0.86 ± 0.12**
*Lamotrigine*	7/7	0.38 ± 0.14	7/7	0.37 ± 0.14	7/7	0.33 ± 0.09
*Carbamazepine*	7/7	0.32 ± 0.07	7/7	0.33 ± 0.07	7/7	0.49 ± 0.08
*Gabapentin*	7/7	0.25 ± 0.10	7/7	0.22 ± 0.09	1/7	0.01 ± 0.01
*Carbamazepine 10.11 epoxide*	7/7	0.07 ± 0.02	7/7	0.05 ± 0.02	7/7	0.04 ± 0.01
**Nonsteroidal anti-inflammatory drugs**	**7/7**	**2.04 ± 0.43**	**7/7**	**1.53 ± 0.27**	**7/7**	**0.11 ± 0.05**
*Diclofenac*	7/7	1.52 ± 0.42	7/7	1.13 ± 0.34	0/7	-
*Tramadol*	7/7	0.25 ± 0.20	7/7	0.24 ± 0.19	7/7	0.11 ± 0.05
*Ketoprofen*	7/7	0.19 ± 0.08	5/7	0.12 ± 0.09	0/7	-
*Niflumic acid*	7/7	0.07 ± 0.03	7/7	0.07 ± 0.03	1/7	0.01 ± 0.01
**Beta-adrenoceptor blocking agents**	**7/7**	**9.44 ± 2.98**	**7/7**	**9.54 ± 3.76**	**7/7**	**1.87 ± 0.54**
*Olmesartan*	7/7	3.73 ± 1.35	7/7	3.78 ± 1.37	7/7	1.36 ± 0.35
*Irbesartan*	7/7	1.98 ± 0.75	7/7	1.97 ± 0.75	7/7	0.14 ± 0.10
*Flecainide*	7/7	0.99 ± 0.22	7/7	1.08 ± 0.38	7/7	0.07 ± 0.03
*Irbesartan 446*	7/7	0.89 ± 0.56	7/7	0.93 ± 0.56	7/7	0.24 ± 0.10
*Valsartan*	7/7	0.55 ± 0.58	7/7	0.64 ± 0.87	0/7	-
*Metoprolol acid*	7/7	0.45 ± 0.23	7/7	0.42 ± 0.24	6/7	0.04 ± 0.01
*Telmisartan*	7/7	0.25 ± 0.15	7/7	0.19 ± 0.11	0/7	-
*Bisoprolol*	7/7	0.15 ± 0.07	7/7	0.14 ± 0.07	3/7	0.02 ± 0.01
*Sotalol*	7/7	0.14 ± 0.04	7/7	0.13 ± 0.04	2/7	0.03 ± 0.01
*Fenofibric acid*	7/7	0.09 ± 0.08	3/7	0.08 ± 0.04	0/7	-
*Metoprolol*	7/7	0.08 ± 0.04	7/7	0.07 ± 0.04	6/7	0.02 ± 0.01
*Atenolol*	7/7	0.08 ± 0.03	7/7	0.06 ± 0.02	2/7	0.02 ± 0.01
*Losartan*	7/7	0.05 ± 0.03	7/7	0.05 ± 0.04	0/7	-
*Clopidogrel*	7/7	0.02 ± 0.01	7/7	0.02 ± 0.01	0/7	-
**UV filters**						
*2-Phenyl-5-benzimidazolesulfonic acid*	7/7	9.50 ± 9.82	7/7	8.93 ± 8.92	2/7	0.46 ± 0.09
**Antipsychotic drugs**	**7/7**	**1.67 ± 0.68**	**7/7**	**1.73 ± 1.03**	**7/7**	**0.60 ± 0.19**
*EDDP*	7/7	0.86 ± 0.42	7/7	1.01 ± 0.77	7/7	0.25 ± 0.14
*Amisulpride*	7/7	0.33 ± 0.19	7/7	0.25 ± 0.14	7/7	0.12 ± 0.05
*Sulpiride*	7/7	0.21 ± 0.07	7/7	0.21 ± 0.07	7/7	0.14 ± 0.04
*Venlafaxine*	7/7	0.19 ± 0.09	7/7	0.18 ± 0.08	7/7	0.09 ± 0.03
*Citalopram*	7/7	0.12 ± 0.04	7/7	0.14 ± 0.03	0/7	-
*Methamphetamine*	7/7	0.07 ± 0.04	6/7	0.09 ± 0.05	0/7	-
**Antihistaminic drugs**						
*Cetirizine*	7/7	0.10 ± 0.04	7/7	0.10 ± 0.04	7/7	0.02 ± 0.02
**Antidiabetic drugs**						
*Sitagliptin*	7/7	0.57 ± 0.12	7/7	0.54 ± 0.21	7/7	0.11 ± 0.06
**X-ray contrast media**						
*Iopromide*	7/7	0.73 ± 1.18	7/7	0.95 ± 1.72	1/7	0.01 ± 0.01

**Table 5:** Primers used for nested RT-PCR for enteric virus amplification.

Virus	Target Region	Primer name	Primer Sequence (5’–3’)	Length (bp)	No. of Cycles	Annealing Temperature (°C)	References
EV	5’ UTR	Ent 1	CGG TAC CTT TGT ACG CCT GT	540	1	55	[64]
		Ent 2	ATT GTC ACC ATA AGC AGC CA				
		neEnt 1	TCC GGC CCC TGA ATG CGG CTA	123	2	55	
		neEnt 2	GAA ACA CGG ACA CCC AAA GTA				
AdV	Hexon	hex1deg	GCC SCA RTG GKC WTA CAT GCA CAT C	301	1	55	[65]
		hex2deg	CAG CAC SCC ICG RAT GTC AAA				
		nehex3deg	GCC CGY GCM ACI GAI ACS TAC TTC	171	2	55	
		nehex4deg	CCY ACR GCC AGI GTR WAI CGM RCY TTG TA				
NoV GI	ORF2	COG1F	CGY TGG ATG CGN TTY CAT GA	381	1	50	[66,67]
		G1-SKR	CCA ACC CAR CCA TTR TAC ATT T				
		G1-SKF	CTG CCC GAA TTY GTA AAT GA	330	2	50	
		G1-SKR	CCA ACC CAR CCA TTR TAC ATT T				
NoV GII	ORF2	COG2F	CAR GAR BCN ATG TTY AGR TGG ATG AG	387	1	52	[66,67]
		G2-SKR	CCR CCN GCA TRH CCR TTR TAC AT				
		G2-SKF	CNT GGG AGG GCG ATC GCA A	344	2	50	
		G2-SKR	CCR CCN GCA TRH CCR TTR TAC AT				
HAV	VP1/2A	2897	TAT TCA GAT TGC AAA TTA YAA T	393	1	40	[68]
		3288	AAY TTC ATY ATT TCA TGC TCC T				
		2949	TAT TTG TCT GTY ACA GAA CAA TCA G	267	2	48	
		3192	AGG RGG TGG AAG YAC TTC ATT TGA				
HEV	ORF1	ORF1F	CCA YCA GTT YAT HAA GGC TCC	348	1	51	[69]
		ORF1R	TAC CAV CGC TGR ACR TC				
		ORF1FN	CTC CTG GCR TYA CWA CTG C	172	2	48	
		ORF1RN	GGR TGR TTC CAI ARV ACY TC				
RoV	NSP3	1950	GCA GTY GTT GYT GYH ACT TCA ACR	986	1	54	[70,71]
		NSP3r	GGT CAC ATA ACG CCC CTA TAG C				
		1958	GTC ATC AGT TGA GTG GTA TCT AAG RT	324	2	52	
		NSP3r	GGT CAC ATA ACG CCC CTA TAG C				
PMMoV	5’ UTR	PMMoV forward	ATG GCT TAC ACA GTT TCC AGT G	474	1	51	[72,73]
		PMMoV reverse	TTA AGG AGT TGT AGC CCA GGT G				
		PMMoVcaps_Fwd	CGT TAG GYA ATC AGT TTC AA	313	2	45	
		PMMoVcaps_Rev2	CGA ACT AAC TCA TTC ATG A				

Legend: enterovirus (EV), adenovirus (AdV), norovirus (NoV), hepatitis A and E (HAV and HEV), rotavirus (RoV). and pepper mild mottle virus (PMMoV).

## References

[1] Tijani J.O., Fatoba O.O., Babajide O.O., Petrik L.F. (2016). Pharmaceuticals, endocrine disruptors, personal care products, nanomaterials and perfluorinated pollutants: A review. Environ. Chem. Lett..

[2] Murgolo S., Yargeau V., Gerbasi R., Visentin F., El Habra N., Ricco G., Lacchetti I., Carere M., Curri M.L., Mascolo G. (2017). A new supported TiO_2_ film deposited on stainless steel for the photocatalytic degradation of contaminants of emerging concern. Chem. Eng. J..

[3] Tran N.H., Reinhard M., Gin K.Y.H. (2018). Occurrence and fate of emerging contaminants in municipal wastewater treatment plants from different geographical regions-a review. Water Res..

[4] Wu J., van Geen A., Ahmed K.M., Alam Y.A.J., Culligan P.J., Escamilla V., Feighery J., Ferguson A.S., Knappett P., Mailloux B.J. (2011). Increase in diarrheal disease associated with arsenic mitigation in Bangladesh. PLoS ONE.

[5] Sousa J.C.G., Ribeiro A.R., Barbosa M.O., Pereira M.F.R., Adrián M.T.S. (2018). A review on environmental monitoring of water organic pollutants identified by EU guidelines. J. Hazard. Mater..

[6] Zhang Y., Kelly W.R., Panno S.V., Liu W.T. (2014). Tracing fecal pollution sources in karst groundwater by Bacteroidales genetic biomarkers, bacterial indicators, and environmental variables. Sci. Total Environ..

[7] Iaconelli M., Muscillo M., Della Libera S., Fratini M., Meucci L., De Ceglia M., Giacosa D., La Rosa G. (2017). One-year surveillance of human enteric viruses in raw and treated wastewaters, downstream river waters, and drinking waters. Food Environ. Virol..

[8] Iaconelli M., Bonanno Ferraro G., Mancini P., Suffredini E., Veneri C., Ciccaglione A.R., Bruni R., Della Libera S., Bignami F., Brambilla M. (2020). Nine-year nationwide environmental surveillance of hepatitis E virus in urban wastewaters in Italy (2011-2019). Int. J. Environ. Res. Public Health.

[9] Mancini P., Bonanno Ferraro G., Suffredini E., Veneri C., Iaconelli M., Vicenza T., La Rosa G. (2020). Molecular detection of human salivirus in Italy through monitoring of urban sewages. Food Environ. Virol..

[10] Masciopinto C., De Giglio O., Scrascia M., Fortunato F., La Rosa G., Suffredini E., Pazzani C., Prato R., Montagna M.T. (2019). Human health risk assessment for the occurrence of enteric viruses in drinking water from wells: Role of flood runoff injections. Sci. Total Environ..

[11] Okeyo A.N., Nontongana N., Fadare Taiwo O., Okoh A.I. (2018). Vibrio Species in Wastewater Final Effluents and Receiving Watershed in South Africa: Implications for public health. Int. J. Environ. Res. Public Health.

[12] La Rosa G., Della Libera S., Iaconelli M., Ciccaglione A.R., Bruni R., Taffon S., Equestre M., Alfonsi V., Rizzo C., Tosti M.E. (2014). Surveillance of hepatitis A virus in urban sewages and comparison with cases notified in the course of an outbreak, Italy 2013. BMC Infect. Dis..

[13] Legislative Decree 3 April 2006. No. 152. Environmental Standards (G.U. n.88 of 14 April 2006). https://www.ecolex.org/details/legislation/legislative-decree-no-152-approving-the-code-on-the-environment-lex-faoc064213/.

[14] Acquedotto Pugliese, Regione Puglia. https://www.aqp.it/node/3.

[15] De Giglio O., Barbuti G., Trerotoli P., Brigida S., Calabrese A., Di Vittorio G., Lovero G., Caggiano G., Uricchio V.F., Montagna M.T. (2016). Microbiological and hydrogeological assessment of groundwater in southern Italy. Environ. Monit. Assess..

[16] De Giglio O., Caggiano G., Bagordo F., Barbuti G., Brigida S., Lugoli F., Grassi T., La Rosa G., Lucentini L., Uricchio V.F. (2017). Enteric viruses and fecal bacteria indicators to assess groundwater quality and suitability for irrigation. Int. J. Environ. Res. Public Health.

[17] Bagordo F., Migoni D., Grassi T., Serio F., Indolo A., Guido M., Zaccarelli N., Fanizzi F.P., De Donno A. (2016). Using the DPSIR framework to identify factors influencing the quality of ground-water in Grecia Salentina (Puglia, Italy). Rend. Lincei Sci. Fis. Nat..

[18] Lugoli F., Leopizzi M.I., Bagordo F., Grassi T., Guido M., De Donno A. (2011). Widespread microbiological groundwater contamination in the South-Eastern Salento (Puglia-Italy). J. Environ. Monit..

[19] Cyprowski M., Stobnicka-Kupiec A., Ławniczek-Wałczyk A., Bakal-Kijek A., Gołofit-Szymczak M., Górny R.L. (2018). Anaerobic bacteria in wastewater treatment plant. Int. Arch. Occup. Environ. Health.

[20] Stevens D.L., Bryant A.E. (2002). The role of clostridial toxins in the pathogenesis of gas gangrene. Clin. Infect. Dis..

[21] Kądzielska J., Obuch-Woszczatyński P., Pituch H., Młynarczyk G. (2012). Clostridium perfringens as the etiological agent of antibiotic associated diarrhoea. Postęp. Microbiol..

[22] Ghorpade K.B., Suryawanshi M., Shinde S.M. (2019). Elimination of Pseudomonas aeruginosa from water systems: A review. J. Biomed. Pharm. Res..

[23] Mena K.D., Gerba C.P. (2009). Risk assessment of Pseudomonas aeruginosa in water. Rev. Environ. Contam. Toxicol..

[24] Bonadonna L., Briancesco R., Suffredini E., Coccia A., Della Libera S., Carducci A., Verani M., Federigi I., Iaconelli M., Bonanno Ferraro G. (2019). Enteric viruses, somatic coliphages and Vibrio species in marine bathing and non-bathing waters in Italy. Mar. Pollut. Bull..

[25] Pushpinder P., Kumar P., Shankar U. (2015). Isolation of Vibrio species on TSA media and comparison of growth percentage of Vibrio species in sea water, pond water, tap water, ground water and river water samples. Int. J. Sci. Res..

[26] Ballal M., Shetty V., Bangera S.R., Prabhu M., Prabhu M. (2017). Vibrio furnissii, an emerging pathogen causing acute gastroenteritis: A case report. JMM Case Rep..

[27] Gerardi M.H. (2006). Wastewater Bacteria.

[28] Oron G., Armon R., Mandelbaum R., Manor Y., Campos C., Gillerman L., Salgot M., Gerba C., Klein I., Enriquez C. (2001). Secondary wastewater disposal for crop irrigation with minimal risks. Water Sci. Technol..

[29] De Giglio O., Quaranta A., Barbuti G., Napoli C., Caggiano G., Montagna M.T. (2015). Factors influencing groundwater quality: Towards an integrated management approach. Ann. Ig..

[30] Goeppert N., Goldscheider N. (2011). Transport and variability of fecal bacteria in carbonate conglomerate aquifers. Ground Water.

[31] Tryland I., Myrmel M., Østensvik Ø., Wennberg A.C., Robertson L.J. (2014). Impact of rainfall on the hygienic quality of blue mussels and water in urban areas in the Inner Oslofjord, Norway. Mar. Pollut. Bull..

[32] Joergensen R.G., Kuntzel H., Scheu S., Seitz D. (1998). Movement of faecal indicator organisms in earthworm channels under a loamy arable and grassland soil. Appl. Soil Ecol..

[33] Xiao H.F., Li G., Li D.M., Hu F., Li H.X. (2014). Effect of different bacterial-feeding nematode species on soil bacterial numbers, activity, and community composition. Pedosphere.

[34] Koumaki E., Mamais D., Noutsopoulos C., Nika M.C., Bletsou A.A., Thomaidis N.S., Eftaxias A., Stratogianni G. (2015). Degradation of emerging contaminants from water under natural sunlight: The effect of season, pH, humic acids and nitrate and identification of photodegradation by-products. Chemosphere.

[35] Denet E., Coupat-Goutaland B., Nazaret S., Pélandakis M., Favre-Bonté S. (2017). Diversity of free-living amoebae in soils and their associated human opportunistic bacteria. Parasitol. Res..

[36] Roberts A.P., Alloy M.M., Oris J.T. (2017). Review of the photo-induced toxicity of environmental contaminants. Comp. Biochem. Physiol. C. Toxicol. Pharmacol..

[37] European Food Safety Authority (EFSA) (2014). Tracing of food items in connection to the multinational hepatitis A virus outbreak in Europe. EFSA J..

[38] Gallone M.F., Desiante F., Gallone M.S., Barbuti G., Tafuri S., Germinario C. (2017). Serosurveillance of hepatitis A in a region, which adopted the universal mass vaccination. Medicine.

[39] Grassi T., Bagordo F., Idolo A., Lugoli F., Gabutti G., De Donno A. (2010). Rotavirus detection in environmental water samples by tangential flow ultrafiltration and RT-nested PCR. Environ. Monit. Assess..

[40] Ruggeri F.M., Bonomo P., Ianiro G., Battistone A., Delogu R., Germinario C., Chironna M., Triassi M., Campagnuolo R., Cicala A. (2015). Rotavirus genotypes in sewage treatment plants and in children hospitalized with acute diarrhea in Italy in 2010 and 2011. Appl. Environ. Microbiol..

[41] Symonds E.M., Nguyen K.H., Harwood V.J., Breitbart M. (2018). Pepper mild mottle virus: A plant pathogen with a greater purpose in (waste) water treatment development and public health management. Water Res..

[42] Rosiles-Gonzalez G., Avila-Torres G., Moreno-Valenzuela O.A., Acosta-Gonzalez G., Leal-Bautista R.M., Grimaldo-Hernandez C.D., Brown J.K., Chaidez-Quiroz C., Betancourt W.Q., Gerba C.P. (2017). Occurrence of pepper mild mottle virus (PMMoV) in groundwater from a karst aquifer system in the Yucatan Peninsula, Mexico. Food Environ. Virol..

[43] Kato R., Asami T., Utagawa E., Furumai H., Katayama H. (2018). Pepper mild mottle virus as a process indicator at drinking water treatment plants employing coagulation-sedimentation, rapid sand filtration, ozonation, and biological activated carbon treatments in Japan. Water Res..

[44] Hamza H., Rizk N.M., Gad M.A., Hamza I.A. (2019). Pepper mild mottle virus in wastewater in Egypt: A potential indicator of wastewater pollution and the efficiency of the treatment process. Arch. Virol..

[45] Gyawali P., Croucher D., Ahmed W., Devane M., Hewitt J. (2019). Evaluation of pepper mild mottle virus as an indicator of human faecal pollution in shellfish and growing waters. Water Res..

[46] Hughes B., Beale D.J., Dennis P.G., Cook S., Ahmed W. (2017). Cross-comparison of human wastewater-associated molecular markers in relation to fecal indicator bacteria and enteric viruses in recreational beach waters. Appl. Environ. Microbiol..

[47] Lee S., Hata A., Yamashita N., Tanaka H. (2017). Evaluation of virus reduction by ultrafiltration with coagulation-sedimentation in water reclamation. Food Environ. Virol..

[48] Castiglioni S., Davoli E., Riva F., Palmiotto M., Camporini P., Manenti A., Zuccato E. (2018). Mass balance of emerging contaminants in the water cycle of a highly urbanized and industrialized area of Italy. Water Res..

[49] Benotti M.J., Brownawell B.J. (2007). Distributions of pharmaceuticals in an Urban Estuary during both dry- and wet-weather conditions. Environ. Sci. Technol..

[50] Musolff A., Leschik S., Schafmeister M.T., Reinstorf F., Strauch G., Krieg R., Schirmer M. (2010). Evaluation of xenobiotic impact on urban receiving waters by means of statistical methods. Water Sci Technol..

[51] Regional Agency for Environmental Protection (ARPA Puglia). https://www.arpa.puglia.it/web/guest/serviziometeo.

[52] Cotecchia V., Calò G.C., Spizzico M., Tinelli R. (1983). Tour in Apulia and Lucania-Geological and Hydrogeological Aspects.

[53] Masciopinto C., Caputo M.C. (2011). Modeling unsaturated-saturated flow and nickel transport in fractured rocks. Vadose Zone J..

[54] De Carlo L., Caputo M.C., Masciale R., Vurro M., Portoghese I. (2020). Monitoring the drainage efficiency of infiltration trenches in fractured and karstified limestone via time-lapse hydrogeophysical approach. Water.

[55] UNI EN ISO 9308-1:2017. Qualità dell’acqua-Conta di Escherichia Coli e Batteri Coliformi-Parte 1: Metodo per Filtrazione su Membrana per Acque Contraddistinte da una Ridotta Flora Batterica di Fondo. http://store.uni.com/catalogo/uni-en-iso-9308-1-2017?josso_back_to=http://store.uni.com/josso-security-check.php&josso_cmd=login_optional&josso_partnerapp_host=store.uni.com.

[56] APAT CNR IRSA 7080, Man 29/2003. Salmonella spp.. http://www.irsa.cnr.it/Docs/Capitoli/7080.pdf.

[57] EN ISO 7899-2, 2003. Water Quality-Detection and Enumeration of Intestinal Enterococci-Part 2: Membrane Filtration Method International Organization for Standardization, Geneva, Switzerland. https://www.iso.org/standard/14854.html.

[58] UNI EN ISO 16266:2008. Water Quality-Detection and Enumeration of Pseudomonas aeruginosa by Membrane Filtration. https://www.iso.org/standard/70091.html.

[59] APAT CNR IRSA 7060 B Man 29 2003. Spore di Clostridi Solfito Riduttori (Acque Superficiali, di Fiume, di Lago, Acque Reflue Anche Sottoposte a Trattamento). http://www.irsa.cnr.it/Docs/Capitoli/7060.pdf.

[60] Noguerola I., Blanch A.R. (2008). Identification of Vibrio spp. with a set of dichotomus keys. J. Appl. Microbiol..

[61] World Health Organization (2003). Guidelines for Environmental Surveillance of Poliovirus Circulation. http://polioeradication.org/wp-content/uploads/2016/07/WHO_V-B_03.03_eng.pdf.

[62] Iaconelli M., Purpari G., Della Libera S., Petricca S., Guercio A., Ciccaglione A.R., Bruni R., Taffon S., Equestre M., Fratini M. (2015). Hepatitis A and E viruses in wastewaters, in river waters, and in bivalve molluscs in Italy. Food Environ. Virol..

[63] BLAST. https://blast.ncbi.nlm.nih.gov/Blast.cgi.

[64] Pina S., Puig M., Lucena F., Jofre J., Girones R. (1998). Viral pollution in the environment and in shellfish: Human adenovirus detection by PCR as an index of human viruses. Appl. Environ. Microbiol..

[65] Allard A., Albinsson B., Wadell G. (2001). Rapid Typing of Human Adenoviruses by a General PCR Combined with Restriction Endonuclease Analysis. J. Clin. Microbiol..

[66] Kageyama T., Kojima S., Shinohara M., Uchida K., Fukushi S., Hoshino F.B., Takeda N., Katayama K. (2003). Broadly reactive and highly sensitive assay for Norwalk-like viruses based on real-time quantitative reverse transcription-PCR. J. Clin. Microbiol..

[67] Kojima S., Kageyama T., Fukushi S., Hoshino F.B., Shinohara M., Uchida K., Natori K., Takeda N., Katayama K. (2002). Genogroup-specific PCR primers for detection of Norwalk-like viruses. J. Virol. Methods.

[68] Taffon S., Bidini G., Vichi F., Corti G., Genovese D., Kondili L.A., Bindi R., Armellini F., Leoncini F., Bartoloni A. (2011). A unique HAV strain circulated in patients with acute HAV infection with different risk exposures in Tuscany, Italy. J. Clin. Virol..

[69] Fogeda M., Avellon A., Cilla C.G., Echevarria J.M. (2009). Imported and autochthonous hepatitis E virus strains in Spain. J. Med. Virol..

[70] Zeng S.Q., Halkosalo A., Salminen M., Szakal E.D., Puustinen L., Vesikaria T. (2008). One-step quantitative RT-PCR for the detection of rotavirus in acute gastroenteritis. J. Virol. Methods.

[71] La Rosa G., Sanseverino I., Della Libera S., Iaconelli M., Ferrero V.E.V., Barra Caracciolo A., Lettieri T. (2017). The impact of anthropogenic pressure on the virological quality of water from the Tiber River, Italy. Lett. Appl. Microbiol..

[72] Chung B.N., Choi H.S., Yang E.Y., Cho J.D., Cho I.S., Choi G.S., Choi S.K. (2012). Tomato spotted wilt virus isolates giving different infection in commercial Capsicum annuum cultivars. Plant Pathol. J..

[73] Colson P., Richet H., Desnues C., Balique F., Moal V., Grob J.J., Berbis P., Lecoq H., Harle J.R., Berland Y. (2010). Pepper mild mottle virus, a plant virus associated with specific immune responses, fever, abdominal pains, and pruritus in humans. PLoS ONE.

[74] Ottaviani M., Bonadonna L. (2007). Rapporti ISTISAN 2007/31. Metodi Analitici di Riferimento per le Acque Destinate al Consumo Umano ai Sensi del D.L. 31/2001.

[75] APAT CNR IRSA 2030 Man 29 2003. Metodi Analitici per le Acque. http://www.irsa.cnr.it/Docs/Capitoli/1000.pdf.

[76] UNI EN 872:2005. Qualità Dell’acqua-Determinazione dei Solidi Sospesi-Metodo per Filtrazione Attraverso Filtri di Fibra di Vetro. http://wwwstore.uni.com/catalogo/uni-en-872-2005.

[77] APHA 22nd ed 2012 5210 D. Respirometric Method. https://beta-static.fishersci.com/content/dam/fishersci/en_US/documents/programs/scientific/technical-documents/white-papers/apha-biochemical-oxygen-demand-white-paper.pdf.

[78] ISO 15705:2002. Water Quality-Determination of the Chemical Oxygen Demand Index (ST-COD)-Small-Scale Sealed-Tube Method International Organization for Standardization, Geneva, Switzerland. https://www.iso.org/standard/28778.html.

[79] UNI 11759:2019. Determinazione dell’azoto Totale-Metodo Mediante Spettrometria UV Dopo Digestione Ossidativa con Persolfato di Sodio Utilizzando una Apparecchiatura Che Opera in Sequenza Analitica Coordinata. http://store.uni.com/catalogo/uni-11759-2019.

[80] UNI EN ISO 10304-1:2009. Qualità Dell’acqua-Determinazione di Anioni Disciolti Mediante Cromatografia Ionica in Fase Liquida-Parte 1: Determinazione di Bromuri, Cloruri, Fluoruri, Nitrati, Nitriti, Fosfati e Solfati. http://store.uni.com/catalogo/uni-en-iso-10304-1-2009.

[81] UNICHIM 2252:2008. Qualità dell’acqua: Determinazione del Fosfato Solubile e del Fosforo Totale-Metodo Colorimetrico Dell’ammonio Fosfoantimonilmolibdato Dopo Sequenza Analitica Coordinata. https://www.unichim.it/metodi/.

[82] Lorimer M.F., Kiermeier A. (2007). Analysing microbiological data: Tobit or not Tobit?. Int. J. Food Microbiol..

[83] Baggi F., Demarta A., Peduzzi R. (2001). Persistence of viral pathogens and bacteriophages during sewage treatment: Lack of correlation with indicator bacteria. Res. Microbiol..

[84] Gerba C.P., Kitajima M., Iker B. (2013). Viral presence in wastewater and sewage and control methods. Viruses in Food and Water Risks, Surveillance and Control.

